# Proton Pump Inhibitor and Tacrolimus Uses are Associated With Hypomagnesemia in Connective Tissue Disease: a Potential Link With Renal Dysfunction and Recurrent Infection

**DOI:** 10.3389/fphar.2021.616719

**Published:** 2021-05-20

**Authors:** Hironari Hanaoka, Jun Kikuchi, Yuko Kaneko, Noriyasu Seki, Hideto Tsujimoto, Kenji Chiba, Tsutomu Takeuchi

**Affiliations:** ^1^ Division of Rheumatology, Department of Internal Medicine, School of Medicine, Keio University, Tokyo, Japan; ^2^ Mitsubishi Tanabe Pharma Corporation, Yokohama, Japan

**Keywords:** proton pump inhibitor, tacrolimus, magnesium, hypomagnesemia, connective tissue disease hypomagnesemia in rheumatology

## Abstract

**Background:** Low levels of serum magnesium perturb renal tubular cell function and lymphocytes, resulting in renal deterioration and an imbalance in mononuclear cells. This study investigated the mechanism and influence of hypomagnesemia in patients with connective tissue disease.

**Methods:** We retrospectively evaluated patients with connective tissue disease and available serum magnesium data who visited Keio University Hospital in 2019. Patients were divided into two groups: those with (serum magnesium < 1.8 mg/dl) and those without hypomagnesemia; their rates of hospitalization for severe infection and cumulative renal deterioration were compared. Patients’ fractions of lymphocytes and natural killer and dendritic cell subsets, as measured by fluorescence-activated cell sorting (FACS) analysis, were also compared.

**Results:** Among 284 patients, hypomagnesemia was detected in 63 (22.2%). Multivariate analysis revealed that the use of proton pump inhibitors [odds ratio (OR), 1.48; *p* = 0.01] and tacrolimus (OR, 6.14; *p* < 0.01) was independently associated with hypomagnesemia. In addition, the renal deterioration rate was significantly higher in tacrolimus and/or proton pump inhibitor users with hypomagnesemia (*p* = 0.01). The hospitalization rate for severe infection was also higher in patients with hypomagnesemia (*p* = 0.04). FACS analysis showed lower CD8+ T cell, CD19+ B cell, natural killer cell, and dendritic cell counts in patients with hypomagnesemia (*p* = 0.03, *p* = 0.02, *p* = 0.02, and *p* = 0.03, respectively).

**Conclusion:** The use of tacrolimus and proton pump inhibitors may be associated with hypomagnesemia and lead to poor renal outcomes and severe infection in patients with connective tissue disease.

## Introduction

Magnesium (Mg) is an abundant intracellular cation that acts as a co-factor for more than 300 enzymes involved in a number of fundamental functions. Mg deficiency leads to many pathogenic conditions, including cardiovascular mortality, stroke, chronic kidney disease (CKD) progression, osteoporosis, and insulin resistance (Mountokalakis, 1990; Kao et al., 1999; He et al., 2006; Ohira et al., 2009; Castiglioni et al., 2013; Al Alawi et al., 2018). Hypomagnesemia is also associated with the development of recurrent infections due to the role of Mg as a second messenger in T cell activation and a contributor to the cytotoxicity of natural killer (NK) cells and CD8+ T cells (Mazur et al., 2007; Li et al., 2011; Chaigne-Delalande et al., 2013). Although the association of hypomagnesemia with wide spectrum disorders has been shown, their causal relationship remains to be proven in most cases.

The causes of hypomagnesemia are categorized into three groups: decreased dietary intake, impaired gastrointestinal absorption, and increased renal loss (Blaine et al., 2015). Several medications are known to influence serum Mg levels through these mechanisms. Although the association between proton pump inhibitors (PPIs) use and development of hypomagnesemia has not been confirmed, PPIs may potentially inhibit pH-dependent active Mg absorption (Perazella, 2013). Calcineurin inhibitors (CNIs) are also associated with low serum Mg concentrations (Barton et al., 1987; Navaneethan et al., 2006), Although both PPIs and CNIs, including tacrolimus (TAC), are frequently used in patients with connective tissue disease (CTD) (Takahashi et al., 2011; Takeuchi et al., 2014; Mok, 2016; Mok et al., 2016; Ueno et al., 2016; Hanaoka et al., 2019; Kaneko et al., 2020; Takada et al., 2020), little is known about their influence on serum Mg levels in the management of CTD.

The aim of this study was to investigate the prevalence of hypomagnesemia and its clinical impact on patients with CTD in relation to PPI and TAC use.

## Materials and Methods

### Patients and the Evaluation of Clinical Data

We reviewed the data of consecutive patients who visited Keio University Hospital from January 2019–December 2019, and were diagnosed with rheumatoid arthritis (RA), systemic lupus erythematosus (SLE), polymyositis/dermatomyositis (PM/DM), Sjögren syndrome (SS), systemic sclerosis (SSc), mixed connective tissue disease (MCTD), anti-neutrophil cytoplasmic antibody-related vasculitis, or other rheumatic diseases according to their respective classification criteria (Bohan and Peter, 1975; Subcommittee for scleroderma criteria of the American Rheumatism Association Diagnostic and Therapeutic Criteria Committee, 1980; Benhamou et al., 1988; Yamaguchi et al., 1992; Hochberg, 1997; Vitali et al., 2002; Taylor et al., 2006; Watts et al., 2007; Aletaha et al., 2010; Dasgupta et al., 2012; Tani et al., 2014; Bashardoust, 2015; Jinnin et al., 2018; Wallace et al., 2020; Heinle and Chang, 2014; Behcet’s Disease Research Committee of Japan.1974). Patients with available serum Mg data were included in the study. Patients who had been treated with oxidized magnesium were excluded. We collected clinical characteristics, treatments administered for more than 3 years, laboratory data, estimated glomerular filtration rate (eGFR) (Levey A.S et al., 2009), and hospitalization due to infection from diagnosis to December 2019. For patients with SLE, we evaluated disease activity using systemic lupus erythematosus activity index (SLEDAI) (Bombardier et al., 1992).

This study was approved by the Ethics Committee of Keio University School of Medicine. Written informed consent was obtained from all subjects prior to blood sample collection as approved by the Institutional Review Board and in accordance with the tenets of the Declaration of Helsinki.

### Definitions

Hypomagnesemia was defined as a serum Mg concentration <1.8 mg/dl (Elgend et al., 2012), and renal deterioration was defined as a >30% elevation in serum creatinine levels from baseline (Badve et al., 2016). Fractional excretion of Mg (FEMg, %) was calculated using the following formula: FE_Mg_ = (U_Mg_ × P_cr_)/(0.7 × P_Mg_ × U_Cr_) × 100 (Agus, 1999), where U and *P* refer to the urine and plasma concentrations of Mg and creatinine (Cr), respectively. Serum Mg concentration was multiplied by 0.7 as only approximately 70% of circulating Mg is free and filters across the glomerulus. The normal limit of FEMg was defined as <2% (Agus, 1999). Severe infection is defined according to previous report (Díaz-Lagares et al., 2011) as those that required intravenous treatment or that led to hospitalization.

### TAC Measurement

TAC concentration was measured using fresh whole blood samples collected 12 h after the last TAC administration by the TACR Flex Dimension immunoassay method using a Dimension EXL analyzer (Siemens Healthcare Diagnostics, Tokyo, Japan) (Takahashi et al., 2011).

### Flow Cytometry

Cell bank blood samples collected at the time of serum Mg measurement from RA patients treated with methotrexate (MTX) monotherapy were analyzed by fluorescence-activated cell sorting (FACS) analysis. Samples were stained with antibodies (BD Biosciences and BioLegend; Supplementary Table S1) and fixed with Phosflow Lyse/Fix Buffer (BD Bioscience). Flow cytometric analysis was conducted on an LSRFortessa^TM^ X-20 (Becton Dickinson) and analyzed using FlowJo ver. 10 (FlowJo, LLC). The phenotypes of immune cell subsets were defined based on the Human Immunology Project protocol (Supplementary Table S2) (Maecker et al., 2012). The mean numbers of immune cell phenotypes were compared.

### Statistical Analyses

Continuous values are shown as the median and interquartile range (IQR). Comparisons between two groups were performed with the Mann-Whitney U-test for continuous variables and the chi-squared test or Fisher’s exact test for categorical variables. The four groups were compared by analysis of variance. Cumulative renal deterioration rates were analyzed using the Kaplan-Meier method with the log-rank test. Correlations between two continuous variables were analyzed using Spearman’s rank correlation coefficient. To identify independent parameters, binary logistic regression analysis was used with variables having a *p*-value < 0.005 in a previous univariate analysis as covariates. A *p*-value < 0.05 was considered statistically significant.

## Results

### Clinical Characteristics

A total of 284 patients with CTD were included in this study. The median (IQR) age was 64.0 (48.0–73.0) years, and 83.8% of the patients were female (Table 1). Underlying CTDs included RA in 108 patients (38.0%), SLE in 59 patients (20.8%), PM/DM in 20 patients (7.0%), SSc in 24 patients (8.5%), MCTD in 10 patients (3.5%), polymyalgia rheumatica in 10 patients (3.5%), microscopic polyangiitis in eight patients (2.8%), IgG4-related disease in nine patients (3.2%), SS in six patients (2.1%), adult Still’s disease in six patients (2.1%), arthritis with palmoplantar pustulosis in six patients (2.1%), eosinophilic granulomatous polyangiitis in four patients (1.4%), psoriatic arthritis in four patients (1.4%), sarcoidosis in two patients (0.7%), Takayasu’s arteritis in two patients (0.7%), granulomatous polyangiitis in two patients (0.8%), Behçet’s disease in two patients (0.8%), diffuse fasciitis in two patients (0.8%), and familial Mediterranean fever in one patient (0.4%). Glucocorticoids were used by 41.5% of all patients, and the median dose was 0.0 (0.0–4.0) mg/day. Among all patients, 141 (49.6%) used PPIs and 68 (23.9%) used TAC. The median dose of TAC was 3.0 (1.5–3.0) mg/day. Hospitalization for severe infection was observed in 25 (8.8%) patients and hypomagnesemia was observed in 63 (22.2%) patients. We next compared Mg levels by underlying diseases (Supplementary Figure S1) and found patients with SLE had the lowest median magnesium level (*p* < 0.001).

**TABLE 1:** Patient characteristics.

	All (*n* = 284)	Normal Mg (*n* = 221)	Hypomagnesemia (*n* = 63)	*p*
Age, years	64.0 (48.0–73.0)	71.0 (51.0–74.0)	55.0 (38.0–69.0)	0.029
Male:Female	46:238	35:186	11:52	0.752
Underlying disease				
RA (%)	108 (38.0)	94 (42.5)	14 (22.2)	
SLE (%)	59 (20.8)	36 (16.3)	23 (36.5)	
PM/DM (%)	20 (7.0)	11 (4.9)	9 (14.2)	
SSc (%)	24 (8.5)	23 (10.4)	1 (1.6)	
MCTD (%)	10 (3.5)	9 (4.1)	1 (1.6)	
PMR (%)	10 (3.5)	7 (3.2)	3 (4.8)	
Others* (%)	53 (18.7)	41 (18.6)	12 (19.0)	
Serum electrolytes				
Mg, mg/dL	2.1 (1.9–2.2)	2.1 (2.0–2.2)	1.7 (1.7–1.8)	<0.001
Na, mEq/L	140.9 (139.7–142.3)	141.2 (139.8–142.4)	140.5 (139.3–141.9)	0.163
K, mEq/L	4.2 (3.9–4.4)	4.2 (4.0–4.4)	4.1 (3.9–4.3)	0.087
CL, mEq/L	105.0 (104.0–107.0)	106.0 (104.0–107.0)	105.0 (103.0–106.0)	0.241
Ca, mEq/L	9.2 (8.9–9.4)	9.1 (8.9–9.4)	9.2 (8.9–9.4)	0.608
P, mEq/L	3.5 (3.2–3.9)	3.5 (3.2–3.9)	3.5 (3.1–3.9)	0.489
Cr, mg/dL	0.71 (0.61–0.83)	0.71 (0.60–0.83)	0.74 (0.63–0.85)	0.477
eGFR, ml/min/1.73m^2^	68.0 (56.0–80.0)	63.0 (56.0–80.0)	70.0 (56.0–81.0)	0.462
Urine markers				
β2-microglobulin, ×10^2^μg/L	1.5 (0.9–2.9)	2.1 (0.9–3.2)	1.2 (0.7–1.9)	0.386
α1-microglobulin, mg/L	3.3 (1.6–6.5)	3.8 (1.6–6.9)	3.3 (1.6–5.8)	0.636
L-FABP, μg/g･Cre	2.4 (1.6–4.7)	3.1 (1.7–4.8)	2.2 (1.4–4.4)	0.164
NAG, IU/L	5.0 (2.5–8.2)	5.0 (2.4–8.1)	5.3 (3.4–9.6)	0.120
NGAL, μg/g･Cre	21.7 (14.0–38.5)	21.7 (15.1–37.1)	21.7 (12.9–46.0)	0.770
Medication				
GC, (%)	118 (41.5)	78 (35.3)	40 (63.4)	0.001
GC dose, median (IQR) mg/day	0 (0–4)	0 (0–3)	3 (0–5)	0.001
TAC (%)	68 (23.9)	34 (15.3)	34 (53.9)	0.001
TAC dose, median (IQR) mg/day	3.0 (1.5–3.0)	2.0 (1.0–3.0)	3.0 (2.5–3.0)	0.037
MMF (%)	13 (4.6)	6 (2.7)	7 (11.1)	0.006
MTX (%)	81 (28.5)	71 (32.1)	10 (15.9)	0.001
AZA (%)	19 (6.7)	17 (7.6)	2 (3.2)	0.213
HCQ (%)	27 (9.5)	13 (5.9)	14 (22.2)	0.001
PPI (%)	141 (49.6)	93 (42.1)	48 (76.1)	0.001
Hospitalization due to infection	25 (8.8)	15 (6.7)	10 (15.8)	0.042
Respiratory infection (%)	18 (6.3)	11 (4.9)	7 (11.1)	0.085
Urinary tract infection (%)	4 (1.4)	2 (1.0)	2 (3.2)	0.607
Skin infection (%)	3 (1.1)	2 (1.0)	1 (1.6)	1.000

Results show median (interquartile range) unless otherwise indicated.

* Others include microscopic polyangiitis, IgG4-related disease, Sjogren’s syndrome, adult Still’s disease, arthritis with palmoplantar pustulosis, eosinophilic granulomatous polyangiitis, psoriatic arthritis, sarcoidosis, Takayasu’s arteritis, granulomatous polyangiitis, Behçet’s disease, diffuse fasciitis, and familial Mediterranean fever.

Mg, magnesium; RA, rheumatoid arthritis; SLE, systemic lupus erythematosus; SSc, systemic sclerosis; MCTD, mixed connective tissue disease; PMR, polymyalgia rheumatica; Cr, creatinine; eGFR, estimated glomerular filtration rate; L-FABP, liver-type fatty acid binding protein; NAG, N-acetyl-β-D-glucosaminidase; NGAL, neutrophil gelatinase-associated lipocalin; GC, glucocorticoid; PPIs, proton pump inhibitors; TAC, tacrolimus; MMF, mycophenolate mofetil; MTX, methotrexate; AZA, azathioprine; HCQ, hydroxychloroquine.

### Factors Associated With Hypomagnesemia

Patients were divided into two groups—the normal Mg group (*n* = 221) and the hypomagnesemia group (*n* = 63)—according to the presence of hypomagnesemia. The clinical characteristics of the two groups were compared (Table 1). The median age was significantly higher in the normal Mg group than in the hypomagnesemia group (71.0 vs. 55.0 years, *p* = 0.029). Renal function including eGFR and serum creatinine levels, electrolyte concentrations (except for Mg), and urine markers were not different between the two groups. Rates of glucocorticoid, PPI, TAC, mycophenolate mofetil (MMF), and hydroxychloroquine (HCQ) use were significantly lower in the normal Mg group than in the hypomagnesemia group (glucocorticoid, 35.3 vs. 63.4%, *p* = 0.001; PPI, 42.1 vs. 76.1%, *p* = 0.001; TAC, 15.3 vs. 53.9%, *p* = 0.001; MMF, 2.7 vs. 11.1%, *p* = 0.006; HCQ, 5.9 vs. 22.2%, *p* = 0.001). The use of MTX was significantly higher in the normal Mg group (32.1 vs. 15.9%, *p* = 0.001).

Hospitalization due to severe infection from the time of CTD diagnosis until December 2019 occurred significantly less often in the normal Mg group than in the hypomagnesemia group (6.7 vs. 15.8%, *p* = 0.042). We additionally compared clinical features between SLE patients with hypomagnesemia and those without (Supplementary Table S3). A higher rate of TAC and PPIs use in SLE patients with hypomagnesemia was also highlighted. Disease activity (SLEDAI) was comparable between SLE patients with hypomagnesemia than SLE patients with normal magnesium (4.0 vs 3.0, *p* = 0.675), but hydroxychloroquine, mycophenolate mofetil, and glucocorticoid were more frequently used in patients with hypomagnesemia (52.2 vs. 36.1%, *p* = 0.284; 21.7 vs. 8.3%, *p* = 0.242; 91.3 vs. 66.7%, *p* = 0.030), suggesting hypomagnesemia could be related with higher disease activity or severity.

Multiple logistic regression analysis identified the use of PPIs [odds ratio (OR) 1.45, 95% confidence interval (CI) 1.0–3.29, *p* = 0.009] and TAC (OR 5.99, 95% CI 2.93–12.24, *p* < 0.001) as independent factors associated with hypomagnesemia (Table 2).

**TABLE 2:** Multivariate analysis for factors associated with hypomagnesemia.

Factor	Odds ratio (95% CI)	P
Age	0.96 (0.95–1.05)	0.152
SLE	1.47 (0.54–3.97)	0.445
RA	0.84 (0.32–2.16)	0.727
GC use	1.14 (0.49–2.71)	0.753
PPI use	1.45 (1.01–3.29)	0.009
TAC use	5.99 (2.93–12.24)	<0.001
MTX use	0.72 (0.27–1.95)	0.523
HCQ use	1.71 (0.53–5.52)	0.371

SLE, systemic lupus erythematosus; RA, rheumatoid arthritis; GC, glucocorticoid; PPIs, proton pump inhibitors; TAC, tacrolimus; MTX, methotrexate; HCQ, hydroxychloroquine.

### Association of Drugs and Mg Levels

**FIGURE 1:**
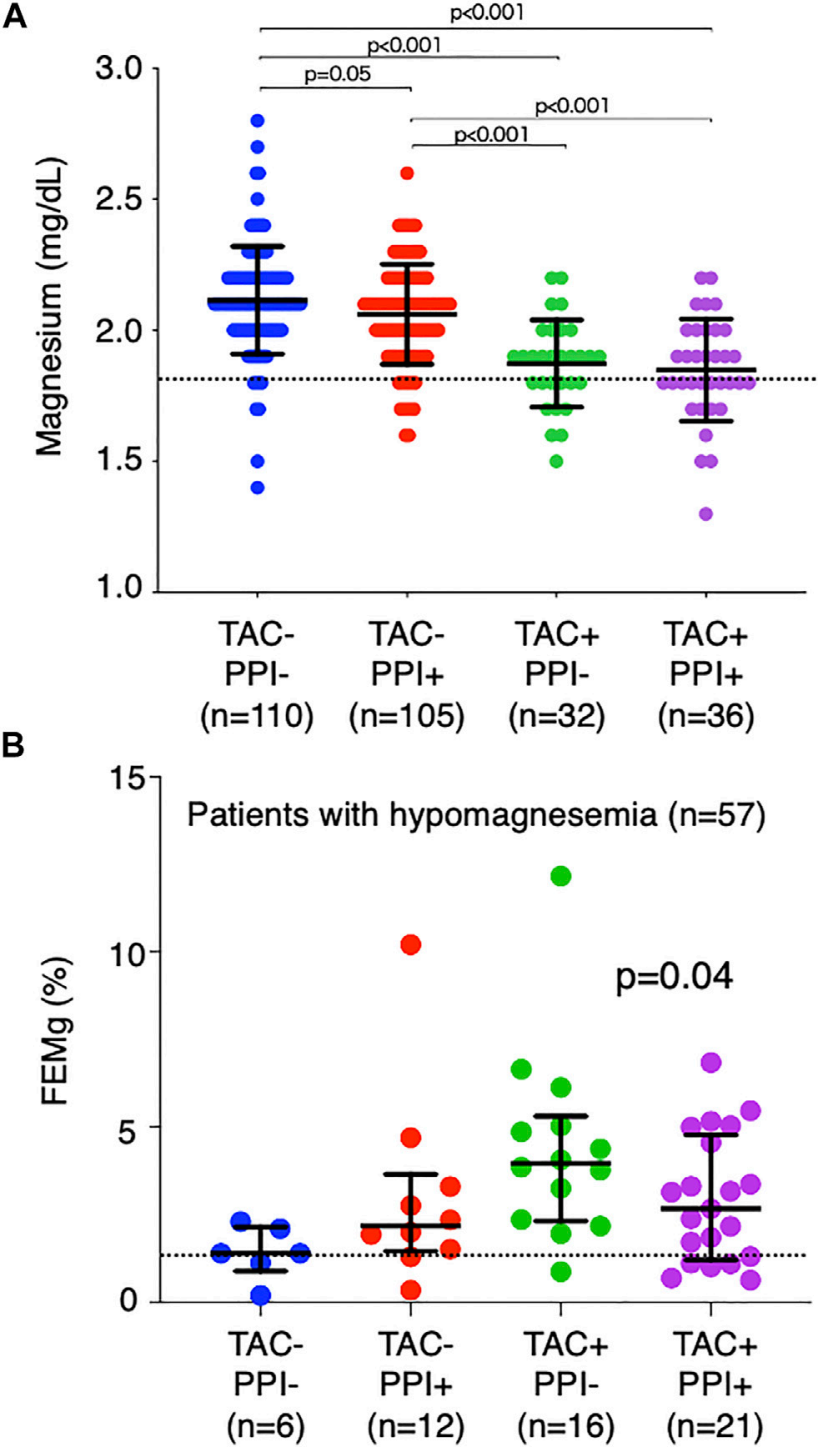
Comparison of magnesium level and fraction excretion of magnesium by drug. All patients were divided into four groups according to TAC and PPI use. **(A)** Comparison of serum Mg levels among the four groups. The dotted line indicates the normal limit of the magnesium level (1.8 mg/dl). **(B)** Comparison of FEMg in patients with hypomagnesemia (*n* = 57). The dotted line indicates the normal limit of FEMg (2.0%). TAC, tacrolimus; PPI, proton pump inhibitor; Mg, magnesium; FEMg, fractional excretion of magnesium.

To further investigate the effects of TAC and PPIs on serum Mg levels, we divided all patients into four groups according to their use of TAC and PPIs (Figure 1A) and compared their Mg levels. Median levels of serum Mg were 2.1 (2.0–2.2) mg/dl in patients that did not use TAC or PPIs, 2.1 (1.9–2.2) mg/dl in those that only used PPIs, 1.9 (1.8–1.9) mg/dl in those that only used TAC, and 1.8 (1.8–2.9) mg/dl in those that used both TAC and PPIs (*p* < 0.0001). When we compared Mg levels in SLE patients, the difference remained (Supplementary Figure S2).

FEMg was then calculated in patients with hypomagnesemia (*n* = 57) (Figure 1B). It was 1.7% (1.5–2.7%) in those that did not use TAC or PPIs, 2.2% (1.6–3.2%) in those that only used PPIs, 3.9% (2.6–4.9%) in those that only used TAC, and 2.7% (1.3–4.7%) in those that used both TAC and PPIs (*p* = 0.04). These findings reflect the different causal mechanisms of hypomagnesemia, namely, that TAC inhibits reabsorption of Mg in the kidneys with a consequent increase in Mg excretion in the urine, while PPIs cause Mg to be wasted in the intestine.

**FIGURE 2:**
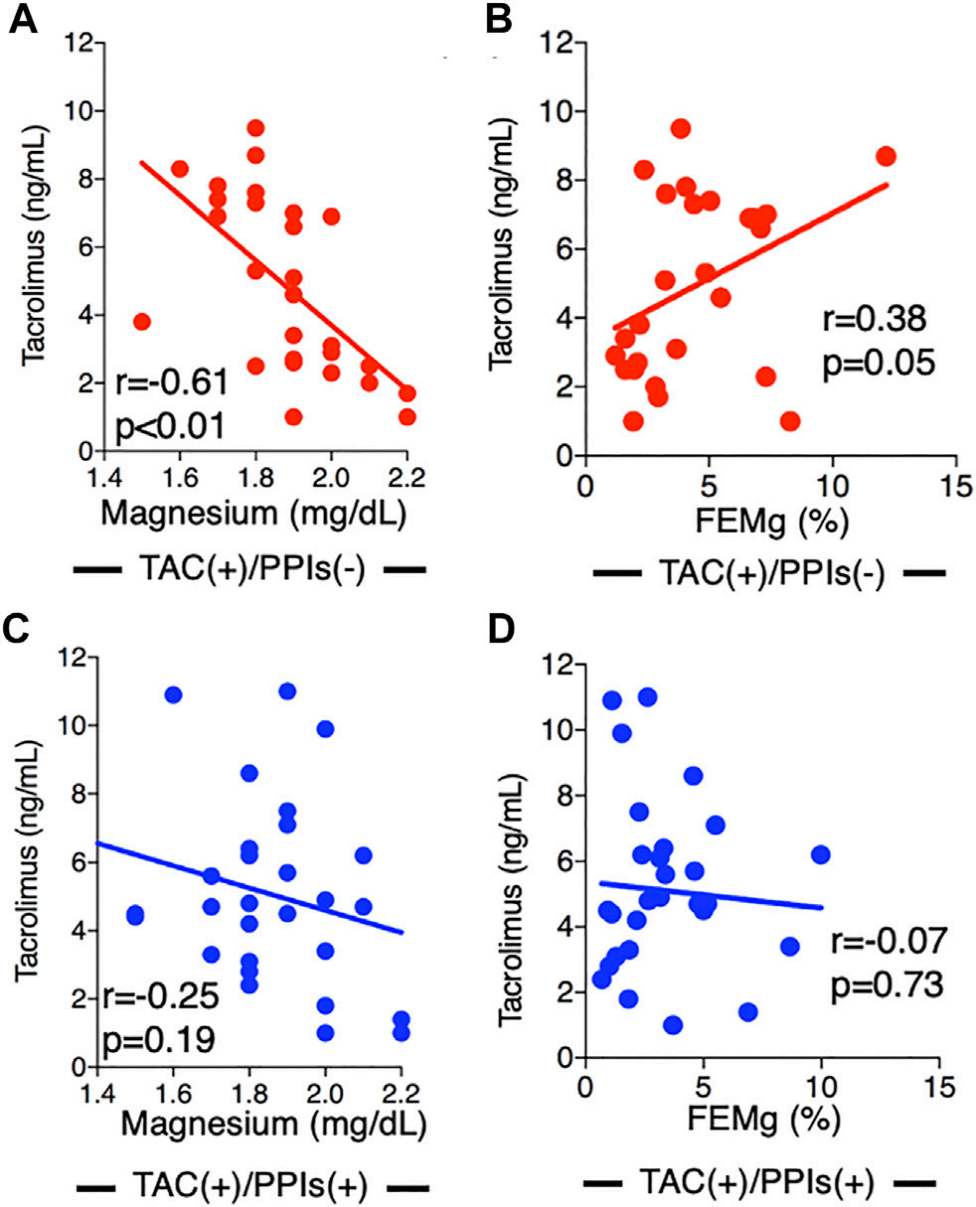
Associations between tacrolimus concentration and magnesium level and tacrolimus concentration and fractional excretion of magnesium by drug. In patients not using PPIs, TAC concentration was significantly correlated with Mg level (r = −0.61, *p* < 0.01) **(A)** and FEMg (r = 0.38, *p* = 0.05) **(B)**. In patients treated with both TAC and PPIs, no association was observed between TAC concentration and Mg level (r = 0.25, *p* = 0.19) **(C)** or FEMg (r = -0.07, *p* = 0.73) **(D)**. PPI, proton pump inhibitor; TAC, tacrolimus; Mg, magnesium; FEMg, fractional excretion of magnesium.

**FIGURE 3:**
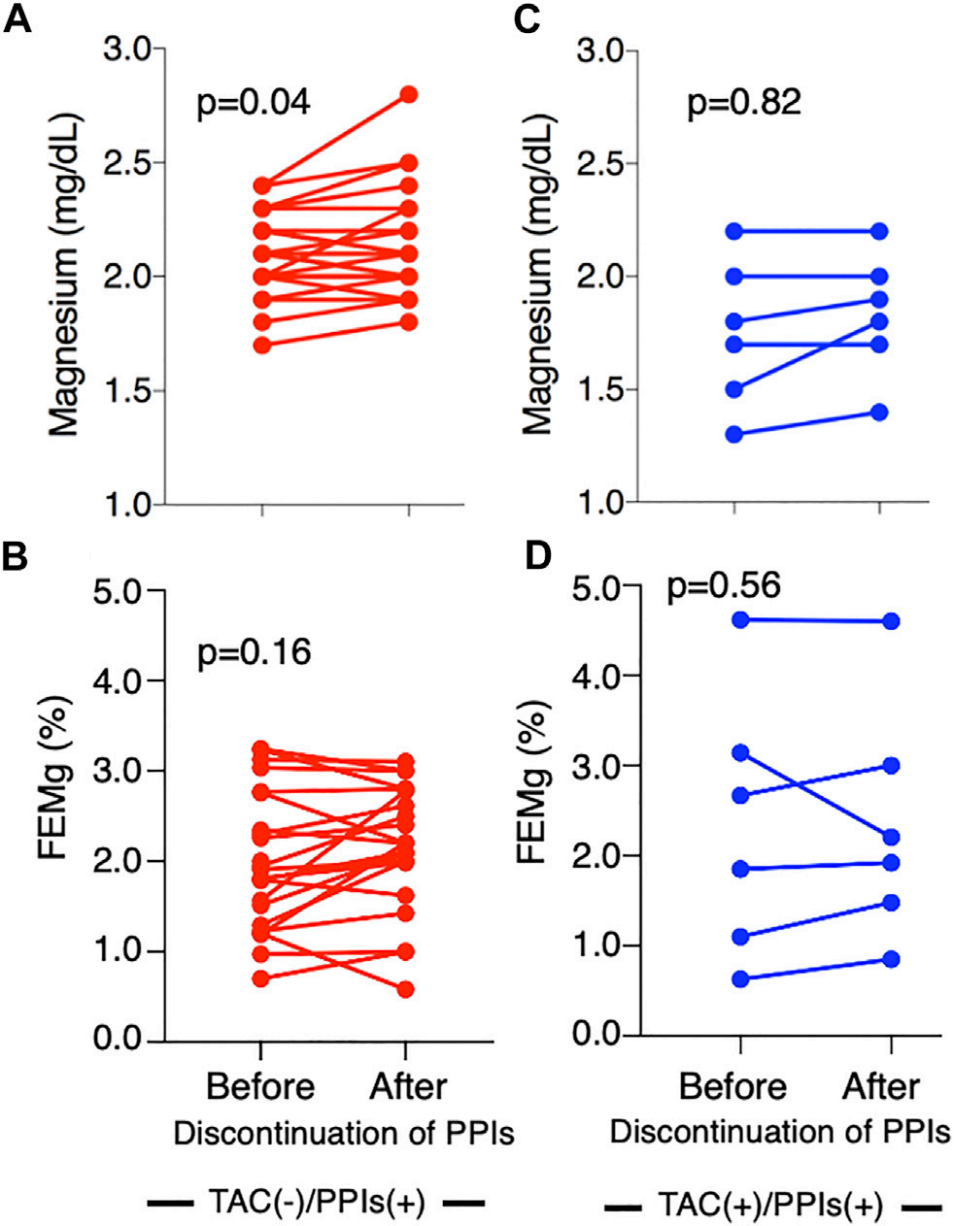
Serial change in magnesium level after the discontinuation of proton pump inhibitors. Mg levels significantly increased after discontinuing PPI use in patients not using TAC (*p* = 0.04) **(A)**; however, no significant difference was seen in FEMg **(B)**. In patients using TAC, no change in Mg level or FEMg was observed after PPI discontinuation **(C,D)**. Mg, magnesium; PPI, proton pump inhibitor; TAC, tacrolimus; FEMg, fractional excretion of magnesium.

The relationship between serum TAC concentrations, serum Mg levels, and FEMg corroborated the effect of the drugs. In patients who did not use PPIs, TAC concentrations were negatively correlated with Mg levels (r = −0.61, *p* < 0.01, Figure 2A) and positively correlated with FEMg (r = 0.38, *p* = 0.05, Figure 2B). In patients who used PPIs, these correlations disappeared (Mg levels, r = −0.25, *p* = 0.19, Figure 2C; FEMg, r = −0.07, *p* = 0.73, Figure 2D). We further investigated 28 patients who had discontinued PPI use at their attending physician’s discretion (without TAC use, *n* = 22; with TAC use, *n* = 6). In patients who did not use TAC, serum Mg levels significantly increased from 2.0 (2.0–2.2) mg/dl to 2.2 (2.0–2.4) mg/dl (*p* = 0.04, Figure 3A) after PPI discontinuation, while FEMg did not change [1.9% (1.4–2.7%) to 2.2% (1.9–2.7%), *p* = 0.16, Figure 3B]. By contrast, in patients treated with TAC, serum Mg concentrations and FEMg did not change when PPI use was discontinued [Mg, 1.8 (1.6–3.0) mg/dl to 2.0 (1.6–4.6) mg/dl, *p* = 0.82, Figure 3C; FEMg, 2.3% (1.3–3.0%) to 2.1% (1.6–0.8%), *p* = 0.56, Figure 3D].

### Relationship Between Hypomagnesemia and Renal Deterioration

**FIGURE 4:**
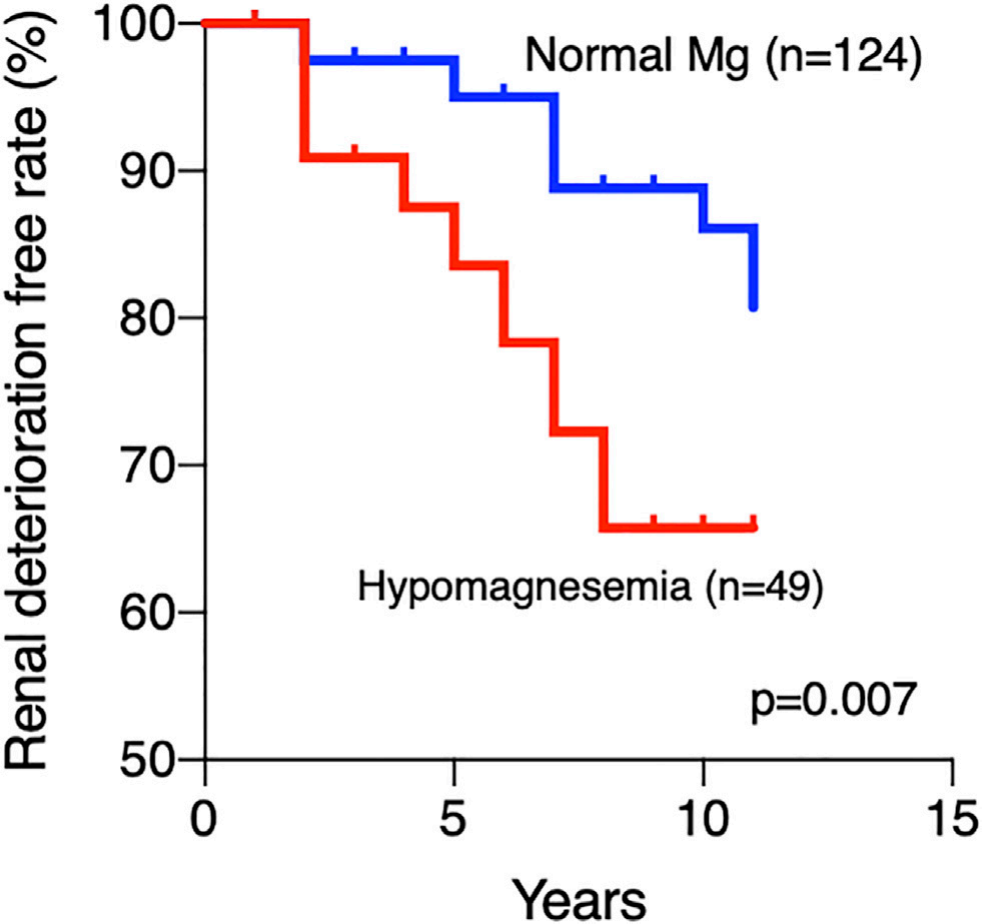
Cumulative renal deterioration-free rate. A significantly lower renal deterioration-free rate was observed in patients with hypomagnesemia than in patients with normal Mg levels (*p* = 0.007) Mg, magnesium.

As the use of TAC and PPIs were the major causes of hypomagnesemia in this study, we investigated the sequential renal function of patients treated with TAC and/or PPIs (*n* = 173) from drug initiation until the last observation. Patients with hypomagnesemia had a higher eGFR than patients with normal Mg at TAC and/or PPIs initiation (82.3 ml/min/1.73 m^2^ vs. 73.4 ml/min/1.73 m^2^, *p* = 0.008). When patients were divided according to the presence of hypomagnesemia, the cumulative renal deterioration-free rates were significantly higher in patients with normal Mg levels (*n* = 124; 80.7%; observation period, 5.0 ± 2.9 years) than in those with hypomagnesemia (*n* = 49; 65.7%; observation period, 5.3 ± 3.4 years) (*p* = 0.007, Figure 4). Furthermore, we selected SLE patients and additionally compared renal deterioration-free rates between those with hypomagnesemia and those without (Supplementary Figure S3). The renal deterioration-free rate in SLE patients with normal Mg levels was higher than in those with normal hypomagnesemia (*p* = 0.019). Of note, renal deterioration was not related to TAC use because renal deterioration-free rates at last observation was 84.5% in the TAC users and 90.1% in the non-TAC users (*p* = 0.34).

### Effect of Hypomagnesemia on Immune Cells

**FIGURE 5:**
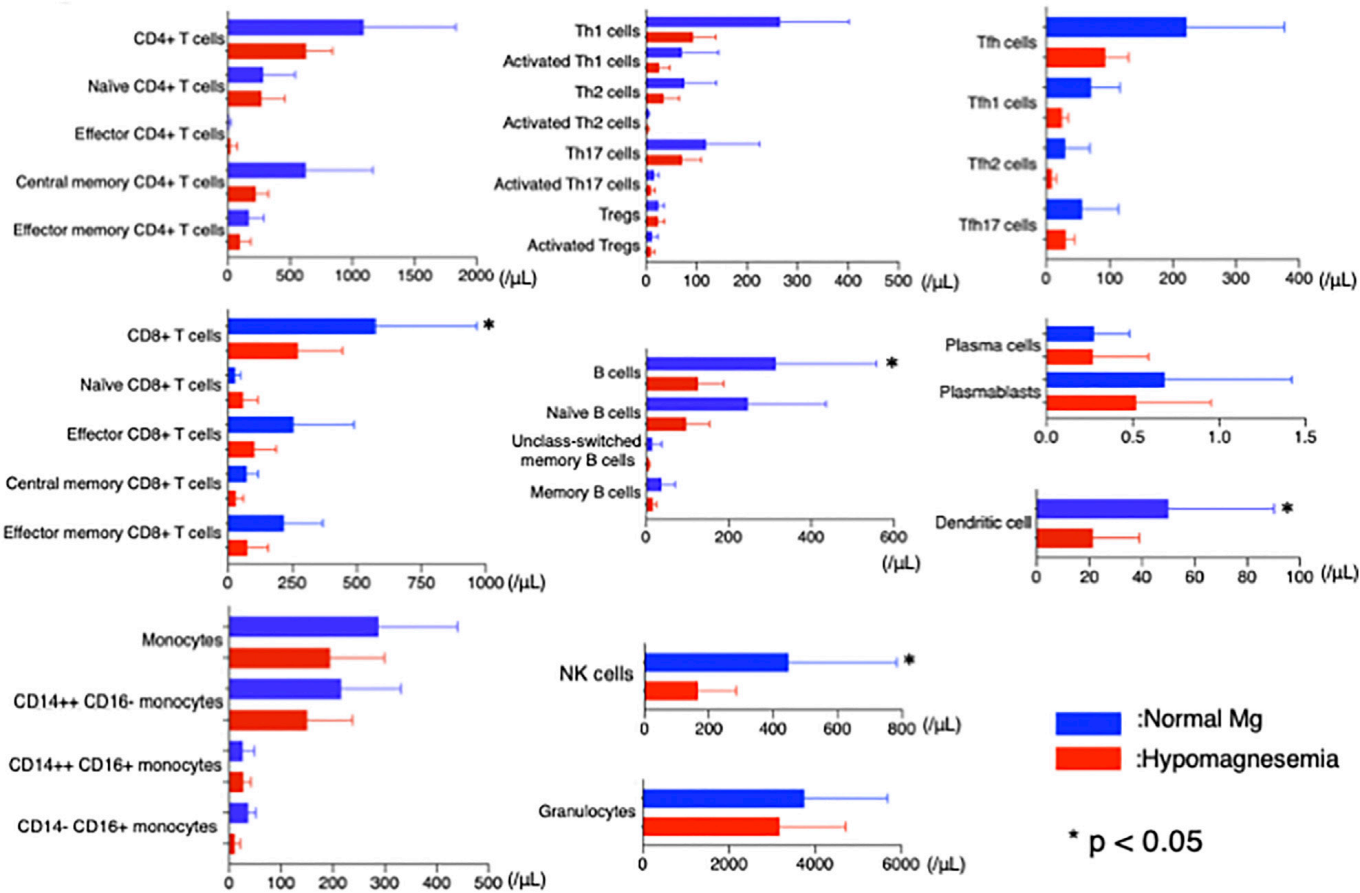
Flow cytometric analysis in patients with and without hypomagnesemia. Lower cells counts were observed for CD8^+^ T cells, CD19^+^ B cells, NK cells, and dendritic cells in patients with hypomagnesemia than in patients with normal Mg levels (*p* = 0.03, *p* = 0.02, *p* = 0.02, and *p* = 0.03, respectively). NK, natural killer; Mg, magnesium.

Among the 283 patients enrolled in this study, 17 patients were concurrently registered in another cohort study at our university. These patients had peripheral blood mononuclear cells available for FACS analysis when their Mg levels were measured. All 17 of these patients had RA and were only treated with MTX. Six of the patients had hypomagnesemia and 11 had normal Mg levels. These patients did not differ with regard to sex, disease duration, disease activity, or MTX dose (Supplemementary Table S4). The number of CD8+ T cells, CD19+ B cells, NK cells, and dendritic cells (DCs) were significantly lower in patients with hypomagnesemia than in patients with normal Mg levels (*p* = 0.03, *p* = 0.02, *p* = 0.02, and *p* = 0.03, respectively, Figure 5). Hospitalization due to infection was observed in one patient with hypomagnesemia (16.6%) and 1 with normal Mg levels (9.1%) (*p* = 0.64).

## Discussion

In this study, hypomagnesemia was observed in approximately 20% of the patients with CTD and was associated with renal deterioration and hospitalization due to severe infection. The development of hypomagnesemia might have been caused by the use of TAC and PPIs.

In addition, a high renal deterioration rate was seen in patients with hypomagnesemia. This finding is consistent with previous studies that have reported the association of hypomagnesemia with incident CKD (Tin et al., 2015), a decline in eGFR in CKD patients (Van Laecke et al., 2013), and the progression to end-stage renal disease in diabetic nephropathy (Sakaguchi et al., 2012). Laecke et al. investigated 1,650 patients with CKD with a median follow-up of 5.1 years and reported that a 1 mg/dl decrease in baseline serum Mg was associated with a 5.1% annual decrease in eGFR. In a study of Japanese patients with diabetic nephropathy (*n* = 144), patients with hypomagnesemia were twice as likely to progress to end-stage renal disease as were those within the normal range. Although the pathogenic mechanism of hypomagnesemia in relation to renal deterioration is not fully understood, hypomagnesemia is considered to damage renal tubules. In one study, incubation of tubular epithelial cells in low-Mg medium increased the rate of apoptosis, whereas this effect was significantly suppressed when Mg concentrations were increased (Sakaguchi et al., 2015).

In the current study, 22.2% of patients with CTDs showed serum Mg levels <1.8 mg/dl (hypomagnesemic). Furthermore, low Mg levels were associated with a high hospitalization rate due to severe infection. The association between hypomagnesemia and recurrent infection was reported in a previous study in which patients with X-linked XMEN, a hereditary immune deficiency syndrome in which dysfunction of the Mg channel, MAGT1, in T lymphocytes leads to a low intra-lymphocytic free Mg concentration, suffered from recurrent infection (Ravell et al., 2014). In another study, hypomagnesemic rats were shown to die earlier than control rats when injected with intravenous *Escherichia coli* endotoxin; however, Mg supplementation improved survival (Salem et al., 1995). In a clinical report on kidney transplantation, low serum Mg was associated with an increased risk of infection, and every 0.1 mg/dl reduction in serum Mg below 2.0 mg/dl increased the hazard ratio by 15% (Van Laecke et al., 2016). Hypomagnesemia decreases T cell count and causes activation and cytotoxicity in CD8+ T cells and NK cells (Gröber, 2019). Although this study did not examine lymphocyte function, we did identify a decrease in the number of CD8+ T cells, CD19+ B cells, NK cells, and DCs in RA patients with hypomagnesemia. Taken together, the decreased function and number of mononuclear cells caused by hypomagnesemia may be associated with impaired immune function in hypomagnesemic patients.

In this study, hypomagnesemia in patients with CTD was significantly associated with the use of TAC and PPIs. These findings are consistent with the action of these drugs, specifically, that TAC interferes with Mg-reabsorption from urine, and PPIs interfere with Mg absorption from the intestines (Barton et al., 1987; Navaneethan et al., 2006; Perazella, 2013). In addition, the interference of TAC with systemic Mg transportation was much greater than that of PPI, as the kidneys can withstand a reabsorption of 20 times more dietary Mg than the intestines (Al Alawi et al., 2018). In fact, our study showed that patients with TAC had lower Mg concentrations than those with PPIs, and the combination of PPIs and TAC did not show additional lowering effect on Mg concentrations than TAC alone. Furthermore, we found that the discontinuation of PPIs increased Mg levels in patients who were not using TAC, suggesting that hypomagnesemia caused by PPIs is reversible. Therefore, we recommend monitoring the serum Mg levels of patients treated with PPIs and considering discontinuation in cases of hypomagnesemia. Mg levels did not change when PPI use was discontinued in patients using both TAC and PPIs. We speculate that this was due to the stronger effect of TAC on lowering serum Mg levels. While we cannot conclude that TAC discontinuation would cause an increase in serum Mg concentrations because no patient discontinued TAC use in our study, based on the findings that TAC concentrations were negatively correlated with serum Mg concentrations, we recommend that TAC dosage be monitored and reduced as much as possible to prevent hypomagnesemia.

Our study has several limitations. First, it was a retrospective, single-center cohort study with a small sample size. This could have caused a degree of selection bias. Second, serum Mg levels were measured cross-sectionally. Therefore, changes in Mg levels during the renal function observation period were unclear. This weakened the discussion regarding the relationship between hypomagnesemia and renal deterioration. Third, PPI use was discontinued at the discretion of attending physicians. This may have also resulted in a degree of selection bias. Fourth, although cyclosporin, one of the calcineurin inhibitors, was used widely in the world, we focused on TAC in this study. The influence of TAC on magnesium level was reported to be greater than that of cyclosporin (Racca et al., 2020), and further investigations may be needed to clarify the difference between the two drugs. Confirmation of our findings will require a multi-center prospective study.

## Conclusion

The use of TAC and PPIs was associated with hypomagnesemia and led to poor renal outcomes and severe infections in patients with CTD. The lowest possible dose of TAC should be prescribed in the management of CTD, and the need for PPIs should be periodically reassessed.

## Data Availability

The original contributions presented in the study are included in the article/Supplementary Material, further inquiries can be directed to the corresponding author.

## Abbreviations

Mg, magnesium; CTD, connective tissue disease; NK, natural killer; DC, dendritic cell; FACS, fluorescence-activated cell sorting; PPIs, proton pump inhibitors; TAC, tacrolimus; CKD, chronic kidney disease; TRPM, transient receptor potential melastatin; CNIs, calcineurin inhibitors; RA, rheumatoid arthritis; SLE, systemic lupus erythematosus; PM/DM, polymyositis/dermatomyositis; SS, Sjögren syndrome; SSc, systemic sclerosis; MCTD, mixed connective tissue disease; eGFR; estimated glomerular filtration rate; FE, fractional excretion; Cr, creatinine; MTX, methotrexate.
