# Supplementary figures and images for: Proton Pump Inhibitor and Tacrolimus Uses are Associated With Hypomagnesemia in Connective Tissue Disease: a Potential Link With Renal Dysfunction and Recurrent Infection
Source: Front Pharmacol. 2021 May 20;12:616719. doi: 10.3389/fphar.2021.616719 (PMC8173076; doi:10.3389/fphar.2021.616719)

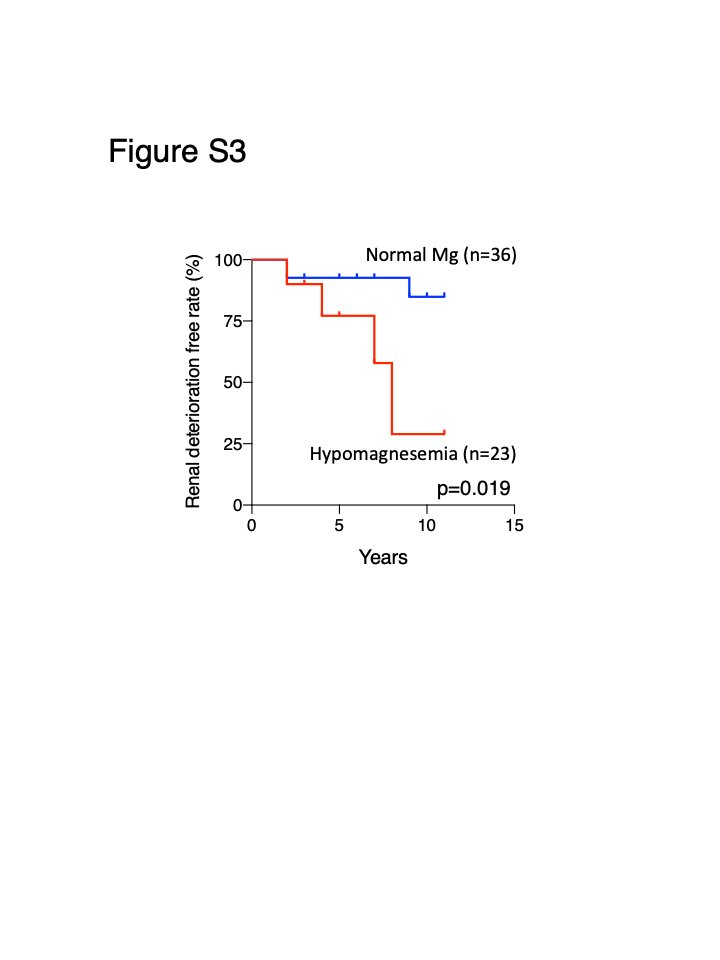

Supplement: Supplementary file 1 [file image3.tiff]

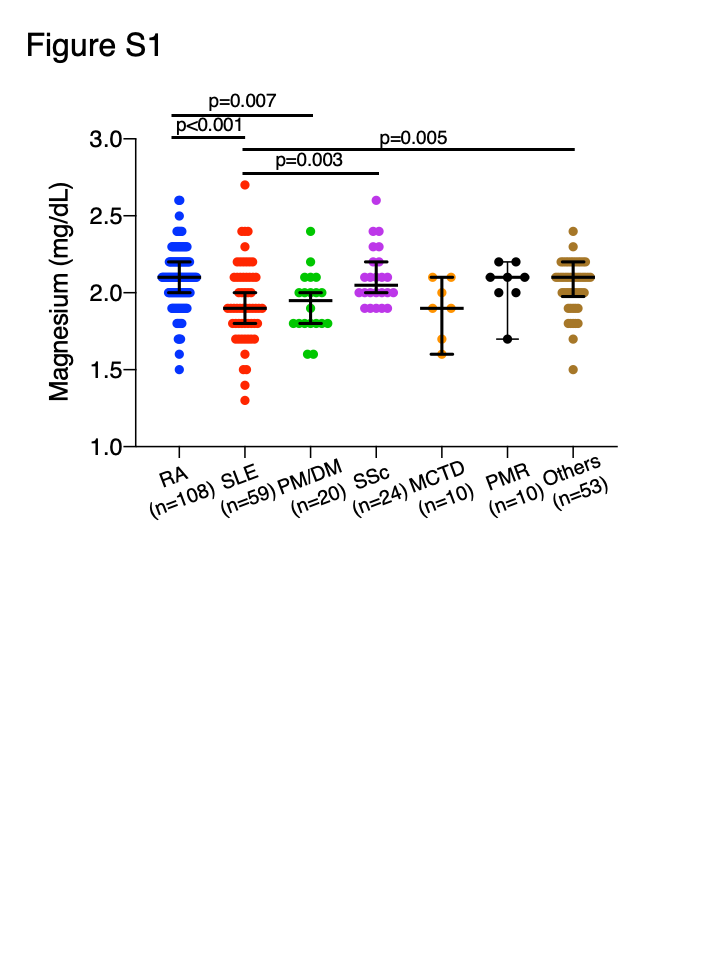

Supplement: Supplementary file 2 [file image1.tiff]

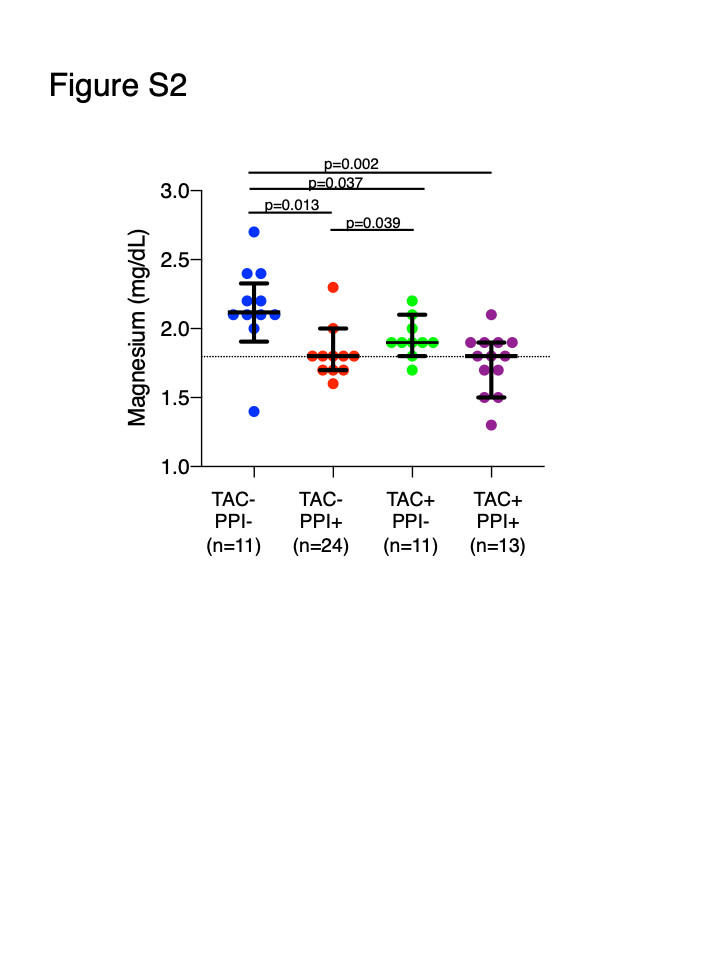

Supplement: Supplementary file 3 [file image2.tiff]
