# Supplementary material for: Proton Pump Inhibitor and Tacrolimus Uses are Associated With Hypomagnesemia in Connective Tissue Disease: a Potential Link With Renal Dysfunction and Recurrent Infection
Source: Front Pharmacol. 2021 May 20;12:616719. doi: 10.3389/fphar.2021.616719 (PMC8173076; doi:10.3389/fphar.2021.616719)
Supplement: Supplementary file 4 [file datasheet1.docx]

Supplementary Material

**Table S1**. Antibodies used in FACS analysis

| Anti-human surface antigen antibody | Fluorescent dye | Clone |
| --- | --- | --- |
| CD3 | FITC | UCHT1 |
| CD3 | PE-CF594 | UCHT1 |
| CD3 | BV786 | SK7 |
| CD4 | BV786 | SK3 |
| CD8 | APC-Cy7 | RPA-T8 |
| CD8 | BV711 | RPA-T8 |
| CD14 | BV605 | M5E2 |
| CD16 | APC | 3G8 |
| CD19 | PerCP-Cy5.5 | HIB19 |
| CD19 | APC-H7 | HIB19 |
| CD20 | APC-H7 | 2H7 |
| CD25 | PE | M-A251 |
| CD27 | PE-Cy7 | M-T271 |
| CD38 | APC | HIT2 |
| CD45RA | PE-Cy7 | HI100 |
| CD45RO | APC-Cy7 | UCHL1 |
| CD56 | FITC | B159 |
| CD127 | FITC | HIL-7R-M21 |
| CD138 | BV605 | MI15 |
| CD183 (CXCR3) | PE | 1C6/CXCR3 |
| CD194 (CCR4) | PE-Cy7 | 1G1 |
| CD194 (CCR4) | BV605 | 1G1 |
| CD196 (CCR6) | BV711 | G034E3 |
| CD197 (CCR7) | PE-CF594 | 150503 |
| CXCR5 | BV421 | RF8B2 |
| HLA-DR | PerCP-Cy5.5 | G46-6 |
| HLA-DR | BV510 | L243 |
| IgD | BV510 | IA6-2 |

**Table S2**. Immuno-phenotyping strategy using antibody staining

| Immune cell subtype | Cell surface marker |
| --- | --- |
| **CD4+ T cell** | CD3+ CD4+ CD8- |
| Naïve CD4+ cell | CD3+ CD4+ CD8- CCR7+ CD45RA+ |
| Effector CD4+ T cell | CD3+ CD4+ CD8- CCR7- CD45RA+ |
| Central memory CD4+ T cell | CD3+ CD4+ CD8- CCR7+ CD45RA- |
| Effector memory CD4+ T cell | CD3+ CD4+ CD8- CCR7- CD45RA- |
| Th1 cell | CD3+ CD4+ CD8- CD45RA- CCR6- CXCR3+ |
| Activated Th1 cell | CD3+ CD4+ CD8- CD45RA- CCR6- CXCR3+ HLA-DR+ |
| Th2 cell | CD3+ CD4+ CD8- CD45RA- CCR6- CXCR3- CCR4+ |
| Activated Th2 cell | CD3+ CD4+ CD8- CD45RA- CCR6- CXCR3- CCR4+ HLA-DR+ |
| Th17 cell | CD3+ CD4+ CD8- CD45RA- CCR6+ CXCR3- |
| Activated Th17 cell | CD3+ CD4+ CD8- CD45RA- CCR6+ CXCR3- HLA-DR+ |
| Treg | CD3+ CD4+ CD45RO+ CCR4+ CD25+ CD127dull CXCR5- |
| Activated Treg | CD3+ CD4+ CD45RO+ CCR4+ CD25+ CD127dull CXCR5- HLA-DR+ |
| Tfh | CD3+ CD4+ CD8- CD45RA- CXCR5+ |
| Tfh1 | CD3+ CD4+ CD8- CD45RA- CXCR5+ CCR6- CXCR3+ |
| Tfh2 | CD3+ CD4+ CD8- CD45RA- CXCR5+ CCR6- CXCR3- CCR4+ |
| Tfh17 | CD3+ CD4+ CD8- CD45RA- CXCR5+ CCR6+ CXCR3- |
| **CD8+ T cell** | CD3+ CD4- CD8+ |
| Naïve CD8+ cell | CD3+ CD4- CD8+ CCR7+ CD45RA+ |
| Effector CD8+ T cell | CD3+ CD4- CD8+ CCR7- CD45RA+ |
| Central memory CD8+ T cell | CD3+ CD4- CD8+ CCR7+ CD45RA- |
| Effector memory CD8+ T cell | CD3+ CD4- CD8+ CCR7- CD45RA- |
| **CD19+ B cell** | CD3- CD19+ |
| Naïve B cell | CD3- CD19+ CD27- IgD+ |
| Unclass-switched memory B cell | CD3- CD19+ CD27+ IgD+ |
| Memory B cell | CD3- CD19+ CD27+ IgD- |
| Plasmablast | CD3- CD19+ CD27+ IgD- CD20- CD38+ CD138- |
| Plasma cell | CD3- CD19+ CD27+ IgD- CD20- CD38+ CD138+ |
| **NK** | CD3- CD19- CD20- CD14- CD56+ HLA-DR- |
| **DC** | CD3- CD19- CD20- CD14- CD56- HLA-DR+ |
| **Monocyte** | CD3- CD19- CD20- CD56- HLA-DR+ CD14+ |
| CD14++ CD16- monocyte | CD3- CD19- CD20- CD56- HLA-DR+ CD14++ CD16- |
| CD14++ CD16+ monocyte | CD3- CD19- CD20- CD56- HLA-DR+ CD14++ CD16+ |
| CD14+ CD16+ monocyte | CD3- CD19- CD20- CD56- HLA-DR+ CD14+ CD16+ |

Th: helper T, Tfh: follicular helper T, NK: natural killer

**Table S3**. Characteristics of patients with systemic lupus erythematosus

|  | All  (n=59) | Normal Mg  (n=36) | Hypomagnesemia  (n=23) | p |
| --- | --- | --- | --- | --- |
| Age, years | 47.0 (37.0–61.0) | 54.0 (44.0–67.0) | 38.0 (29.0–52.0) | 0.038 |
| Male:Female | 3:56 | 2:34 | 1:22 | 1.012 |
| SLEDAI | 4.0 (2.0-7.0) | 3.0 (2.0-5.0) | 4.0 (2.0-5.0) | 0.675 |
| Serum electrolytes |  |  |  |  |
| Mg, mg/dL | 1.9 (1.8–2.1) | 2.1 (1.9–2.2) | 1.7 (1.7–1.8) | <0.001 |
| Na, mEq/L | 140.6 (139.2–142.1) | 141.2 (139.4–142.2) | 140.2 (139.1–141.1) | 0.412 |
| K, mEq/L | 4.0 (3.8–4.3) | 4.0 (3.8–4.3) | 4.0 (3.8–4.2) | 0.884 |
| CL, mEq/L | 106.0 (104.3–107.0) | 106.0 (104.0–107.0) | 105.0 (104.0–106.0) | 0.322 |
| Ca, mEq/L | 9.1 (8.9–9.4) | 9.0 (8.9–9.3) | 9.1 (8.9–9.4) | 0.266 |
| P, mEq/L | 3.6 (3.2–4.1) | 3.6 (3.4–4.1) | 3.6 (3.1–4.1) | 0.735 |
| Cr, mg/dL | 0.74 (0.63-0.85) | 0.73 (0.62-0.86) | 0.74 (0.65-0.80) | 0.465 |
| eGFR, ml/min/1.73m^2^ | 68.0 (58.0–81.0) | 68.0 (55.0–81.0) | 70.0 (61.0–81.0) | 0.351 |
| Urine markers |  |  |  |  |
| β2-microglobulin, ×10^2^μg/L | 1.5 (0.8–2.9) | 2.0 (1.0–4.0) | 1.3 (0.7–2.2) | 0.124 |
| α1-microglobulin, mg/L | 3.7 (1.5–5.6) | 4.5 (1.6–7.9) | 3.3 (1.6–5.8) | 0.436 |
| L-FABP, μg/g･Cre | 2.4 (1.6–5.3) | 2.9 (1.8–6.3) | 3.0 (1.4–4.7) | 0.271 |
| NAG, IU/L | 5.6 (3.3–10.3) | 5.3 (2.9–10.3) | 5.6 (5.3–10.1) | 0.213 |
| NGAL, μg/g･Cre | 21.7 (12.0–50.4) | 21.7 (11.6–47.3) | 26.7 (13.1–50.8) | 0.366 |
| Medication |  |  |  |  |
| GC, (%) | 45 (76.3) | 24 (66.7) | 21 (91.3) | 0.030 |
| GC dose, median (IQR) mg/day | 3 (1–6) | 2.5 (0–5) | 4 (2–6) | 0.591 |
| TAC (%) | 24 (40.7) | 9 (25.0) | 15 (65.2) | 0.003 |
| TAC dose, median (IQR) mg/day | 3.0 (1.5-3.0) | 2.5 (1.5-3.0) | 3.0 (2.5-3.0) | 0.037 |
| MMF (%) | 8 (13.6) | 3 (8.3) | 5 (21.7) | 0.242 |
| MTX (%) | 4 (6.8) | 3 (8.3) | 1 (4.3) | 1.000 |
| AZA (%) | 5 (8.5) | 4 (11.1) | 1 (4.3) | 0.639 |
| HCQ (%) | 25 (42.4) | 13 (36.1) | 12 (52.2) | 0.284 |
| PPI (%) | 37 (62.7) | 18 (50.0) | 19 (82.7) | 0.014 |
| Hospitalization due to infection | 5 (8.5) | 2 (5.6) | 3 (13.0) | 0.367 |

Results show median (interquartile range) unless otherwise indicated.

*Others include microscopic polyangiitis, IgG4-related disease, Sjogren’s syndrome, adult Still’s disease, arthritis with palmoplantar pustulosis, eosinophilic granulomatous polyangiitis, psoriatic arthritis, sarcoidosis, Takayasu’s arteritis, granulomatous polyangiitis, Behçet’s disease, diffuse fasciitis, and familial Mediterranean fever.

Mg, magnesium; SLE, systemic lupus erythematosus; SLEDAI, systemic lupus erythematosus activity index; Cr, creatinine; eGFR, estimated glomerular filtration rate; L-FABP, liver-type fatty acid binding protein; NAG, N-acetyl-β-D-glucosaminidase; NGAL, neutrophil gelatinase-associated lipocalin; GC, glucocorticoid; PPIs, proton pump inhibitors; TAC, tacrolimus; MMF, mycophenolate mofetil; MTX, methotrexate; AZA, azathioprine; HCQ, hydroxychloroquine

**Table S4**. RA patients with or without hypomagnesemia

|  | Normal Mg  (n=11) | Hypomagnesemia  (n=6) | p |
| --- | --- | --- | --- |
| Age (Year) | 72.0 (68.0-79.0) | 63.0 (47.0-71.5) | 0.06 |
| Male: Female | 1 : 10 | 0 : 6 | 0.44 |
| Disease duration (months) | 102.0 (75.0-129.0) | 60.0 (48.0-138.0) | 0.89 |
| Mg (mg/dL) | 2.3 (2.1-2.3) | 1.9 (1.7-1.9) | < 0.01 |
| CDAI | 1.8 (1.2-2.1) | 2.0 (0.7-5.1) | 0.65 |
| Lymphocytes (/μL) | 3021.3  (2358.5-3060.6) | 1270.3  (958.2-1741.8) | 0.08 |
| CRP (mg/dL) | 0.03 (0.03-0.11) | 0.05 (0.02-0.10) | 0.27 |
| RF (IU/mL) | 22.5 (19.0-66.5) | 112.0 (18.0-153.0) | 0.83 |
| Anti-CCP (U/mL) | 100.0 (52.9-115.8) | 6.9 (0.6-24.4) | 0.70 |
| Treatment |  |  |  |
| MTX (%) | 11 (100) | 6 (100) | - |
| MTX dose (mg/week) | 8 (6.5-8.0) | 8.0 (6.0-10.0) | 0.96 |
| TAC (%) | 0 (0) | 0 (0) | - |
| GC (%) | 0 (0) | 0 (0) | - |
| bDMARDs | 0 (0) | 0 (0) | - |
| PPI (%) | 6 (54.5) | 4 (66.7) | 0.62 |
| Hospitalization (%) | 1 (9.1) | 1 (16.6) | 0.64 |

RA, rheumatoid arthritis; Mg, magnesium; CDAI, Clinical Disease Activity Index; CRP, C-reactive protein; RF, rheumatoid factor; CCP, cyclin citrullinated peptide; MTX, methotrexate; TAC, tacrolimus; GC, glucocorticoid; bDMARDs, biological disease-modifying anti-rheumatic drugs; PPI, proton pump inhibitor
